# Obstetrics and Gynecology

**Published:** 1894-05

**Authors:** 


### SELECTIONS AND ABSTRACTS.

### GYNECOLOGY AND OBSTETRICS.

EDITED BY DR. VIRGIL O. HARDON.

### PUERPERAL ECLAMPSIA.

Puerperal eclampsia has been the subject of many recent investigations.

Gerdes (*Centralblatt für Gynäkologie*, 1892, No. 20) gives the results of bacteriological investigations of the lungs, blood, liver, and kidneys of women who have died of eclampsia. He found bacteria resembling the bacillus of chicken cholera. Injected into mice these germs produce convulsions, coma and death, and the same events follow their injection into rats, guinea-pigs, rabbits, and pigeons. He concludes that puerperal eclampsia is due to a specific micro-organism found in the blood and tissues of women suffering from this disease.

Hofmeister (*Fortschritte der Medicin*, 1892, Nos. 22 and 23) made experiments with bacilli which he obtained from Gerdes, and declares these bacilli to be the same as the proteus vulgaris or common bacterium of putrefaction.

Haegler (*Centralblatt für Gynäkologie*, 1892, No. 15) made experiments with the urine of eclampsia patients, and in one case the streptococcus pyogenes albus was found, but the patient had chronic nephritis. In a fatal case the urine and tissues contained the diplococcus pneumoniæ. He does not support Gerdes's views.

Fehling and Döderlein (*Centralblatt für Gynäkologie*, 1892, No. 51, and 1893, No. 1) are also against the views of Gerdes, and agree with Dührssen that rapid delivery is the best mode of treatment.

Dührssen (*Archiv für Gynäkologie*, 1893) has given a full and exhaustive account of about two hundred cases of eclampsia, with their treatment and results. For the latest information this paper should be read in full in the original. His chief points are the following: Puerperal eclampsia is due most frequently to blood intoxication. Kreatin and kreatinin are freely present in the blood from non-elimination due to the kidneys in pregnancy, chronic nephritis, hydronephrosis, hyperæmia of the kidneys, and to retention of urine from pressure on the ureters. Centers in the cortex of the brain capable of producing coma and convulsions are excited by the kreatin and kreatinin (Landois). An excitable condition of the nervous centers aids these causes, and in some cases convulsive attacks may be caused by reflex excitation of the cortical centers. [Sir William Roberts, in his book on urinary diseases, gives the substance of these conclusions in a less developed condition.] A fatty degeneration of the kidneys, heart, stomach, mucous membranes, and destruction of the red blood cells is found in severe cases, and chloroform inhalation, when prolonged, increases this tendency. Each attack of eclampsia increases the danger. Emptying the uterus produces cessation of the eclampsia in 93.75 per cent of the cases, and therefore delivery is indicated at once. The mortality is much higher in cases which are allowed to terminate naturally. To aid delivery he uses Barnes's bags, and in certain cases deep incisions of the cervix. He strongly objects to chloroform inhalations, if prolonged, and other than to aid speedy delivery, and chloral hydrate and morphine are also forbidden.

---

### ICE IN PHLEGMASIA ALBA DOLENS.

Dr. John A. Miller (*Pacific Med. Jonrnal*), in treating on the subject of "milk leg," speaks highly of the efficacy of the cold treatment of the disease. He first used it in 1886, and since then has used it in six cases with uniform and decided success. The procedure was in the following manner: An ordinary large towel was dipped into iced water, wrung out and clapped around the affected limb; a heavy flannel roller bandage was then applied from the toes upward to the groin. On the most painful parts, like the inner aspect of the thigh, the popliteal region and the calf of the leg, were laid rubber bags filled with ice. These were kept in place by a circular binder, independent and outside of the roller bandage. The patient was a little shocked when the cold towel was first applied, but the unpleasantness was only momentary, and then the reaction brought ease and comfort. She desired the ice bags to be renewed quite often at first, as she claimed they relieved the pain, as anything else had never done before. The pain was entirely controlled by the cold. The temperature dropped from 103° to 100° the next day, and the patient commenced to improve, which continued uninterruptedly. The towel was freshly dipped from four to six times in the twenty-four hours. As soon as the patient experienced relief, she was quite anxious to endure the temporary chill from a fresh compress, because the limb felt always better for it afterward. The towels soon became dry and hot, and this gave rise to painful symptoms again.—*St. Louis Medical and Surgical Journal.*

---

### MARRIAGE, DYSMENORRHEA AND HYSTERIA.

Wythe Cook (*Amer. Jour. of Obstet.*, December, 1893) finds from experience that in most cases of dysmenorrhea and hysteria amongst single women marriage aggravates the disease. Hysteria is by no means cured by marriage, dysmenorrhea often returns after pregnancy. One patient suffered from very severe dysmenorrhea. She married, on advice, but the disease was aggravated by coitus. Conception occurred, and she fully believed that pregnancy would cure her, but the menstrual pain returned immediately after weaning. Another patient, subject to dysmenorrhea, married when twenty and became pregnant when over twenty-three. She bore a healthy child, and then took to the morphine habit. Her husband died a few months after her confinement. The period was suppressed for five years. After she ceased to take morphine it reappeared, at first irregularly, and at length in due season, but in both cases there was severe pain. She married again, and has remained eighteen months sterile; the dysmenorrhea continues.

A young woman subject to headaches and hysterical manifestations attended with hallucinations and depression, got married. The neuroses were not improved by marriage. A robust young lady, free from hysteria, married and bore two children within twenty-one months after marriage. Hysterical swoonings occurred during the pregnancies. A patient subject to dysmenorrhea and hysterical fits married and bore five children. The menstrual pain never reappeared after the first pregnancy, but the fits still occur.—*Ontario Med. Jour.*

---

MERZ: RUPTURE OF THE UTERUS: ITS TREATMENT (*Archiv. für Gynäkologie*, Band xlv., Heft 2).

The author reports *in extenso* two cases of ruptured uterus. One was treated expectantly and died; the other, which was operated on, terminated in recovery. To obtain a good picture of the various methods of treatment and their results, he collected two hundred and thirty cases out of the current literature. In one hundred and eighty-one of these a complete rupture of the uterus was noted. Rupture of the uterus is observed under widely differing conditions, and various factors may be the exciting cause. Therefore Merz found that, even through so large a collection of cases, he was unable to arrive at final conclusions, and to a certain extent we must individualize the treatment in each individual case. The author favors operative treatment, although in his statistics the expectant plan (iodoform wick and uterine drainage) gives the best results, namely, 66.6 per cent. recovery, as compared with 48.1 per cent. from laparotomy.

Should the child be *in utero*, he advises delivery *per vias naturales;* in all other cases immediate laparotomy and closure of the uterine wound, or the Porro operation.

If laparotomy must be excluded, drainage of the uterus and packing of the wound with the iodoform wick, without preceding irrigation, is advocated.

---

HOW TO DRESS THE CORD.

The cord may easily be dressed without the use of the binder in the following manner:

Supposing that the cord has been safely ligated and severed, a small wad of antiseptic absorbent cotton is wrapped about its entire length, the surrounding surface of skin having previously been thoroughly cleansed in the bath and afterwards dried; the cord is then bound to the abdomen by a small strip of adhesive plaster, one inch wide and six long. The whole affair is allowed to remain in this condition until the time arrives for the cord to have separated, when the plaster is easily removed by applying, for a few minutes, a towel wet with warm water. Should there be any further need of dressing the same process can be easily reapplied.

The simplicity, cleanliness and ease with which this method of treating the cord is employed at once makes obvious its superiority over the old method of dressing in doing away with the dangerous binder.

A clean, healthy-looking wound will be left after the cord has separated, which, with a second small pad of antiseptic cotton applied for a week longer, insures a satisfactory healing of these parts.—*Times and Register.*

---

### PREMATURE LABOR.

Pelzer (*Archiv für Gynäkologie,* No. 42, Part 2,) gives the method used in the Cologne Maternity Hospital for producing premature labor, and also in cases where the labor pains are feeble and ineffective. This consists in injecting about five ounces of glycerine between the membranes and the uterine wall by means of a syringe with a long curved nozzle. The syringe must be freed of air bubbles. Uterine contractions are set up by direct irritation of the uterine surface, by the mechanical separation of the membranes from the uterine walls, and by causing transudation of the liquor amnii owing to the hygroscopic qualities of glycerine, the ovum is lessened, and the further separation of the membranes results. Its action is certain and quick, and the glycerine is antiseptic, and lubricates the genital canal.—*Fred Edge.*

---

### MOTHER'S MILK NOT STERILE.

Few things can be regarded as being quite pure and untainted nowadays, and our readers will not be surprised to hear that mother's milk must be numbered with the majority. Honigmann finds that human milk obtained with all antiseptic precautions from seventythree breasts of sixty-four nursing women was only sterile when duly cultivated in four cases. In the remainder staphylococcus albus was present, and in forty-four cases the staphylococcus aureus; while in three instances other bacteria, a bacillus, and a sarcina were found. The number of germs varied from one to upward of 9,000 in a cubic millimetre. These observations are of interest in reference to the occurrence of thrush in children, to the origin of which they may furnish a clew, and also to the liability ,that children while still sucking present to suppuration after wounds accidentally or intentionally inflicted.—*Lancet.*

